# Comments on the model parameters in “SiFit: inferring tumor trees from single-cell sequencing data under finite-sites models”

**DOI:** 10.1186/s13059-019-1692-5

**Published:** 2019-05-16

**Authors:** Hamim Zafar, Anthony Tzen, Nicholas Navin, Ken Chen, Luay Nakhleh

**Affiliations:** 1 Department of Computer Science, Rice University, Houston, Texas USA; 2 Department of Bioinformatics and Computational Biology, the University of Texas M.D. Anderson Cancer Center, Houston, Texas USA; 3 Department of Genetics, the University of Texas M.D. Anderson Cancer Center, Houston, Texas USA

## Derivation of matrix *Q* in Equation (5) in Methods

Each site in the (diploid) genome can be in one of five states, 0/0, 0/-, 0/1, 1/-, or 1/1, where 0 denotes reference allele, 1 denotes variant allele, and - denotes a deletion.

We introduce two rate parameters:

- *λ*_*d*_: the rate of recurrent point mutation at a site.
- *λ*_*l*_: the combined rate of deletion and loss of heterozygosity (LOH).

Using these five states and rate parameters, we have the following instantaneous rate matrix *Q*:

We now abstract the genotypes as follows:

- Genotype 0 corresponds to states 0/- and 0/0.
- Genotype 1 corresponds to state 0/1.
- Genotype 2 corresponds to states 1/- and 1/1.

Under this abstraction and the assumptions detailed in the caption of Table 1, we obtain the matrix *Q* given in Eq. (5) in the main text.

**Table 1:** Expanded *Q* matrix for ternary data

	0/-	0/0	0/1	1/-	1/1
0/-	0	NA	NA	NA	NA
0/0	*λ*_*l*_	-1 - *λ*_*l*_	1	NA	NA
0/1	$\frac {\lambda _{l}}{2}$	$\frac {\lambda _{d}}{2}$	−(*λ*_*d*_+*λ*_*l*_)	$\frac {\lambda _{l}}{2}$	$\frac {\lambda _{d}}{2}$
1/-	NA	NA	NA	0	NA
1/1	NA	NA	*λ*_*d*_	*λ*_*l*_	−*λ*_*l*_−*λ*_*d*_

*Q*(*i*,*j*) denotes transition from state *i* to state *j*. The transitions for which the entry is ‘NA’, are not allowed. In particular, we do not allow the transitions 0/- → 1/- or 1/- →0/- as a reflection of a simplifying assumption that a recurrent point mutation and deletion/LOH occurring at the same site is a very rare event. Furthermore, we do not model copy number gain.

The following clarifications also apply to the main text:

- Given the explanation above, the following statement should be removed: “LOH events can result in the genotype transitions 1→0 and 1→2 whereas deletions can result in the genotype transitions 1→0,1→2 or 2→1. To compute the infinitesimal rates for these transitions, we introduce two parameters *λ*_*d*_ and *λ*_*l*_ that account for the effects of deletion and LOH respectively.”
- The statement
  “It is important to note that out of the three different types of events that could hint at a deviation from the infinite-sites assumption, SiFit currently models events (deletions, LOH, etc.) that affect the same genomic site more than once and the FP and FN errors in SCS data.”
should be replaced by
  “It is important to note that out of the three different types of events that could hint at a deviation from the infinite-sites assumption, SiFit currently models events (recurrent point mutations, deletions, LOH, etc.) that affect the same genomic site more than once and the FP and FN errors in SCS data.”
- The statement
  “These parameters being relative quantities (they denote the rates of deletion and LOH, respectively, relative to the rate of point mutations), we choose a beta distribution as their prior.”
should be replaced by
  “These parameters being relative quantities (they denote the rates of recurrent point mutation and deletion/LOH, respectively, relative to the rate of point mutations), we choosea beta distribution as their prior.”

